# Adenomyosis Accompanied by Multiple Hemorrhagic Cerebral Infarction: A Case Report

**DOI:** 10.7759/cureus.59280

**Published:** 2024-04-29

**Authors:** Bin Chi, Meiyan Liu, Pengwei Hou, Jianwu Wu, Shousen Wang

**Affiliations:** 1 Neurosurgery, Fuzhou 900th Hospital, Fuzong Clinical Medical College, Fujian Medical University, Fuzhou, CHN; 2 Gastroenterology, Fuzhou 900th Hospital, Fuzong Clinical Medical College, Fujian Medical University, Fuzhou, CHN

**Keywords:** women and health, hemorrhagic transformation, cerebral hemorrhage, cerebral infarction, adenomyosis

## Abstract

This study aims to present a case of uterine adenomyosis accompanied by multiple hemorrhagic cerebral infarctions (CIs), summarize therapeutic experiences based on the literature review, and improve the clinical diagnosis and treatment of multiple hemorrhagic CIs. This paper describes a 46-year-old female with a four-year history of uterine adenomyosis complicated by multiple hemorrhagic CIs. During treatment, elevated levels of D-dimer, CA-125, and severe anemia were observed. Following internal medicine treatment targeting uterine adenomyosis and hemorrhagic CIs, the cerebral hemorrhage gradually resolved. Women presenting with multiple CIs, particularly hemorrhagic ones, should be evaluated for the presence of gynecological diseases. Treating gynecological conditions may aid in the management of multiple CIs.

## Introduction

Uterine adenomyosis is a benign gynecological condition characterized by the infiltration of endometrial tissue into the uterine myometrium, with the endometrial tissue composed of glands and stroma [1-3]. Adenomyosis commonly occurs in women of childbearing age and is often associated with symptoms such as dysmenorrhea, menorrhagia, and heavy menstrual bleeding (HMB); although approximately 30% of women with adenomyosis are asymptomatic, the prevalence of adenomyosis diagnosed by imaging is estimated to be 20-30% [3,4].

In some patients, adenomyosis may progress to cerebral infarctions (CIs), particularly in middle-aged women with severe anemia and elevated levels of D-dimer and CA-125. Reports suggest that women with adenomyosis who have elevated levels of glycoprotein CA-125 and D-dimer, especially during menstruation, are at higher risk of acute CI [5]. However, to our knowledge, there have been few reports of adenomyosis complicating multiple hemorrhagic CIs. This study aims to improve the clinical diagnosis and treatment of multiple hemorrhagic CIs by presenting a case of adenomyosis accompanied by multiple hemorrhagic CIs and summarizing treatment experiences based on a literature review.

## Case presentation

**Figure 1:**
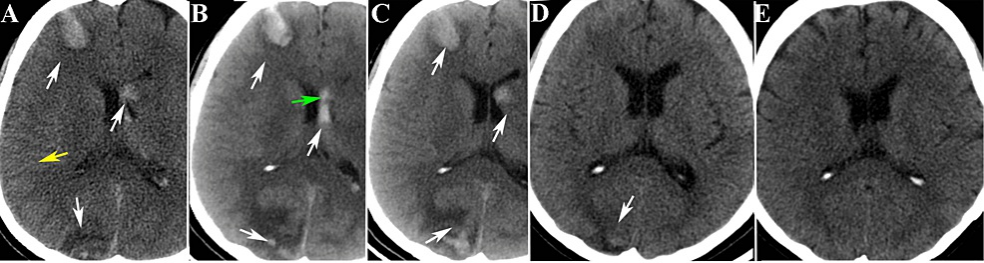
The basal ganglia level images of cranial CT scans before and after the onset of the illness A shows the cranial CT upon admission to the neurosurgery department (yellow arrow indicating subarachnoid hemorrhage and the white arrow indicating hemorrhagic focus); B depicts the cranial CT image on the first day of admission (green arrow indicating hemorrhage from the thalamus into the ventricle and the white arrow indicating aggravation of the hemorrhagic focus); C displays the cranial CT scan performed on the seventh day after admission (white arrow indicating slight absorption of the hemorrhagic focus compared to previous images); D presents the cranial CT scan taken two weeks after admission (white arrow indicating old hemorrhagic focus); E exhibits the cranial CT scan during the patient's orthopedic hospitalization.

A 46-year-old woman was admitted to the neurosurgery department due to a one-day history of headaches and recurrent seizures with altered consciousness for one hour. She experienced persistent headaches one day prior to admission and subsequently developed altered consciousness, generalized muscle stiffness, limb convulsions, and foaming at the mouth one hour before admission, with her eyes deviating to the right. Her last menstrual period occurred on June 27, 2023, and headaches developed after menstruation. She had a history of increased menstrual bleeding for 10 years and was diagnosed with uterine fibroids five years ago. There was no history of hypertension, diabetes, heart disease, hyperlipidemia, or family history of cerebrovascular diseases. Additionally, she had not taken contraceptives or hormone medications. A cranial CT scan revealed hemorrhagic CI in the right occipital lobe, multiple hemorrhages in the right frontal lobe, left basal ganglia, left thalamus, and left lateral ventricle, along with subarachnoid hemorrhage (Figure 1A).

**Figure 2:**
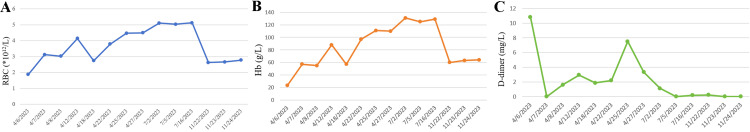
Line chart of some laboratory indexes of patients regularly reviewed during hospitalization A, B, and C show numerical line plots of red blood cells, Hb, and D-dimer, respectively. Hb, hemoglobin

One day later, there was a progression of intracranial hemorrhage, with the appearance of hemorrhagic foci around the infarcted area (Figure 1B). The lumbar puncture revealed red-colored cerebrospinal fluid with a pressure of 320 mmH_2_O. Hemoglobin (Hb) levels were normal (Figure 2A), while D-dimer levels were elevated (Figure 2C) , and serum CA-125 levels were elevated ( 253 U/mL ).

**Figure 3:**
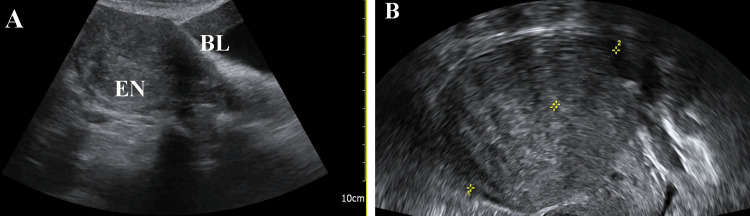
Ultrasound images of the uterus A shows the uterine ultrasonography; the uterus appears uniformly enlarged and symmetrical (68.8×64.3×55.7 mm) with a thickened muscle layer and heterogeneous echo, and the endometrium is centrally located with unclear demarcation from the muscle layer. B - The muscular layer of the anterior wall was thicker than that of the posterior wall, about 42.1 mm (yellow crosshair 1) and 29.3 mm (yellow crosshair 2) EN, endometrium; BL, bladder

Three months prior to admission, the patient sustained a tibial fracture due to a fall and underwent open reduction and internal fixation of the tibial fracture at our orthopedic department. Significant decreases in Hb levels and elevated D-dimer levels were noted before the surgery ( Figures 2B, 2C ). After receiving symptomatic treatments such as blood transfusion and anticoagulation, orthopedic surgery was performed. Color Doppler ultrasound revealed a thrombus in the left posterior tibial vein (14x4.5 mm), and gynecological ultrasonography showed uterine enlargement (78x73x64 mm, Figure 3A). The muscular layer of the anterior wall was thicker than that of the posterior wall, about 42.1 mm (yellow crosshair 1) and 29.3 mm (yellow crosshair 2), and the echo of the muscular layer was uneven (Figure 3B). Subsequently, anticoagulant therapy with nadroparin calcium (1 mL - 9500 IU) was administered at a dose of 4250 IU, 85 IU/kg (0.1 mL/kg) subcutaneously daily, along with a transfusion of suspended red blood cells to improve Hb levels ( Figure 2).​​​​​

**Figure 4:**
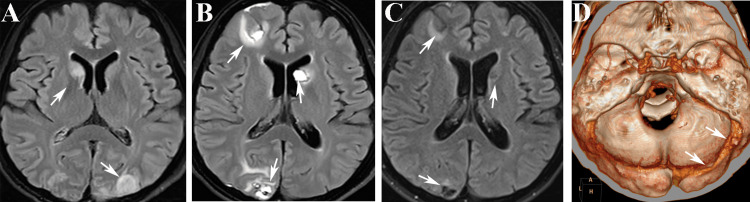
The MRI T2 flair images and cervical CTV obtained during the course of the illness A shows the MRI image taken during the first seizure episode while hospitalized in the orthopedic department (white arrows indicate ischemic lesions in the right caudate nucleus head and left occipital lobe). B displays the MRI findings two weeks after the hemorrhage (white arrows indicate ischemic lesions with necrosis in the right frontal lobe, occipital lobe, and left thalamus). C presents the follow-up cranial MRI conducted one month later (white arrows indicate old hemorrhagic lesions). D exhibits the three-dimensional reconstruction image of the cervical CTA (white arrows indicate two filling defects in the right transverse sinus). CTV, CT venogram

During April 2023 , while hospitalized in the orthopedic ward, the patient experienced three episodes of grand mal seizures, characterized by generalized muscle stiffness, upper limb convulsions, frothing at the mouth, and transient loss of consciousness. A comprehensive cranial MRI scan revealed multiple infarct lesions in the bilateral parietal and occipital lobes, as well as the right caudate nucleus (Figure 4A). So we considered at that time that the patient had secondary epilepsy due to multiple intracranial infarctions. The dynamic electrocardiogram did not reveal paroxysmal atrial fibrillation. Additionally, no significant abnormalities were observed in the dynamic electroencephalogram or the cranial CT scan (Figure 1E). After discharge from the orthopedic ward, the patient was prescribed oral sodium valproate ( 500 mg bid ) to control epilepsy. During the neurosurgery inpatient period in July 2023, following conservative treatments such as lumbar puncture to release hemorrhagic cerebrospinal fluid and mannitol to reduce intracranial pressure, the patient experienced improvement in symptoms such as headache and altered consciousness. Subsequent imaging on the seventh and 14th days showed gradual absorption of the hematoma (as depicted in Figures 1C, 1D, 4B, and 4C). Regarding the treatment for adenomyosis, due to the patient's refusal of definitive hysterectomy, she opted for standardized therapy with gonadotropin-releasing hormone agonists (GnRH-α) ( GnRH-α injection, leuprorelin acetate, 3.75 mg, subcutaneous injection, every 1/4 week, administered a total of three times on 2023-5-16, 2023-6-22, 2023-7-10) .

The patient was discharged three weeks later, maintaining regular oral administration of sodium valproate () to control seizures. Additionally, she received standardized treatment for adenomyosis with GnRh-α at the gynecology outpatient clinic. A follow-up examination after one month revealed a right transverse sinus thrombosis on the neck CT venogram (CTV)(Figure 4D). During the follow-up period, there were no reported symptoms of headache or seizures, and levels of Hb and D-dimer remained within the normal range (Figure 2C).

## Discussion

We report a case of a 46-year-old female with adenomyosis and multiple hemorrhagic CIs, who presented with elevated D-dimer, CA-125, and severe anemia during hospitalization. According to the literature, the mechanisms underlying adenomyosis-induced multiple CIs may be associated with factors such as elevated D-dimer, elevated CA-125, severe anemia, and menstrual periods, leading to hypercoagulability, hormone replacement therapy, and increased tissue factor (TF) levels [1]. To the best of our knowledge, this is the first report of adenomyosis complicated by multiple hemorrhagic CIs.

Although reports of adenomyosis complicated by multiple CIs are rare, similar cases have been documented. These occurrences may be associated with the following factors: (1) elevated D-dimer is a reliable predictor of ischemic stroke, as its elevation signifies thrombin formation and fibrinolysis [2]. Ohara indicated that D-dimer can be utilized for etiological classification and diagnosis of ischemic stroke, while Kim et al. similarly suggested that admission D-dimer >1.0 mg/L is an independent predictor of recurrent ischemic stroke [3,4]. (2) Elevated CA-125 levels may increase the risk of ischemic stroke. Previous studies have reported that elevated CA-125 is associated with a higher risk of ischemic stroke [5]. CA-125 is a biomarker used for monitoring epithelial ovarian cancer and for distinguishing pelvic masses. Previous reports have also documented elevated CA-125 levels in patients with adenomyosis who experienced concurrent stroke [5]. This cancer antigen, CA-125, induces systemic thromboembolism by activating platelets and neutrophils, leading to clot formation throughout the body [6]. Therefore, elevated CA-125 levels are believed to be associated with a hypercoagulable state. (3) In patients with adenomyosis, the elevated immunoreactive expression of TF in the endometrial tissue plays a significant role in the coagulation cascade [7,8]. (4) Anemia may also be a risk factor for thrombus formation. (5) Iron deficiency is a common cause of anemia, and iron deficiency anemia has been linked to thrombotic events in cancer patients [9,10]. (6) Hemostatic dysfunction resulting from menstrual bleeding during the menstrual period is also considered a potential risk factor for CI [11]. In this case, the patient also presented with elevated D-dimer and CA-125 levels, and the lowest Hb level reached 23g/L. Despite intermittent blood transfusions and conservative medical treatment, the patient still had mild to moderate anemia. Therefore, the CIs observed in this patient were likely related to the aforementioned two factors.

In this case, the woman was admitted to the neurosurgery department due to intracerebral hemorrhage rather than CI. We speculate that this may be related to hemorrhagic transformation (HT). It has been reported that approximately 10% of stroke patients experience HT [12]. According to the time of occurrence, HT can be classified into two types: early HT occurs within several days (within five days) after acute CI, while delayed HT occurs in the subacute phase (approximately one week to one month) [13]. On one hand, the mechanism of HT after CI is related to the loss of basal membrane components of brain microvessels and decreased vascular wall integrity following infarction [13,14]. On the other hand, HT is also significantly associated with petechiae near the damaged vessels and the development of bleeding [15]. Risk factors for HT after CI include advanced age, greater stroke severity, elevated blood glucose, atrial fibrillation, INR >1.7, congestive heart failure, renal impairment, and dual antiplatelet therapy [15,16]. Dual antiplatelet therapy also increases the risk of HT [17]. The reason for HT in this case could be the worsening severity of the stroke. However, there might also be other factors yet to be discovered, as the patient was not on dual antiplatelet therapy and did not have any other aforementioned risk factors. Small hemorrhages have minimal impact on clinical symptoms and prognosis, while large hemorrhages can affect thrombolytic and anticoagulant therapy, with severe cases potentially leading to impaired consciousness or death. Surgical treatment is not routine for HT, and in this patient with multiple bleeding sites, conservative treatment was chosen. After the disappearance of neurological symptoms through rehabilitative exercises, the patient was discharged. Upon follow-up, a CTV revealed thrombosis in the right transverse sinus, but as the patient did not exhibit significant clinical symptoms, and to prevent further bleeding, continued anticoagulant therapy was not initiated.

According to literature reports, the treatment of adenomyosis plays a crucial role in cases of adenomyosis combined with multiple CIs [18]. Surgical treatment for adenomyosis, such as hysterectomy, may be an effective method for preventing recurrent CIs [1]. However, in this case, the patient declined surgical treatment and opted for GnRH-α therapy for adenomyosis. Based on the follow-up CTV results showing thrombosis in the right transverse sinus, we consider that GnRH-α therapy alone still carries a risk of recurrent CIs, which aligns with the viewpoint of Zhao [19]. To prevent the patient from experiencing further stroke risks, it is recommended that the patient seek proper treatment for adenomyosis from the gynecology department, including surgical intervention such as hysterectomy.

## Conclusions

This study reports a case of a 46-year-old woman with adenomyosis complicated by multiple hemorrhagic CIs. The diagnostic and treatment process of this case suggests that women with multiple hemorrhagic CIs accompanied by elevated D-dimer, CA-125, and anemia should be assessed for concurrent gynecological diseases. This includes not only malignant tumors such as ovarian cancer but also benign conditions such as adenomyosis. Furthermore, early treatment of gynecological diseases after hemorrhage stabilization is crucial to prevent stroke recurrence. Surgical treatment for adenomyosis may be a preferable preventive measure.
